# Medical Student Performance on the National Board of Medical Examiners Emergency Medicine Advanced Clinical Examination and the National Emergency Medicine M4 Exams

**DOI:** 10.5811/westjem.2015.9.27305

**Published:** 2015-11-12

**Authors:** Katherine Hiller, Joseph House, Luan Lawson, Stacey Poznanski, Thomas K. Morrissey

**Affiliations:** * University of Arizona School of Medicine, Department of Emergency Medicine, Tucson, Arizona; † University of Michigan School of Medicine, Department of Emergency Medicine, Ann Arbor, Michigan; ‡ East Carolinas University Brody School of Medicine, Department of Emergency Medicine, Greenville, North Carolina; § Wright State University School of Medicine, Department of Emergency Medicine, Dayton, Ohio; ¶ University of Florida School of Medicine-Jacksonville, Department of Emergency Medicine, Jacksonville, Florida

## Abstract

**Introduction:**

In April 2013, the National Board of Medical Examiners (NBME) released an Advanced Clinical Examination (ACE) in emergency medicine (EM). In addition to this new resource, CDEM (Clerkship Directors in EM) provides two online, high-quality, internally validated examinations. National usage statistics are available for all three examinations, however, it is currently unknown how students entering an EM residency perform as compared to the entire national cohort. This information may help educators interpret examination scores of both EM-bound and non-EM-bound students.

**Objectives:**

The objective of this study was to compare EM clerkship examination performance between students who matched into an EM residency in 2014 to students who did not. We made comparisons were made using the EM-ACE and both versions of the National fourth year medical student (M4) EM examinations.

**Method:**

In this retrospective multi-institutional cohort study, the EM-ACE and either Version 1 (V1) or 2 (V2) of the National EM M4 examination was given to students taking a fourth-year EM rotation at five institutions between April 2013 to February 2014. We collected examination performance, including the scaled EM-ACE score, and percent correct on the EM M4 exams, and 2014 NRMP Match status. Student t-tests were performed on the examination averages of students who matched in EM as compared with those who did not.

**Results:**

A total of 606 students from five different institutions took both the EM-ACE and one of the EM M4 exams; 94 (15.5%) students matched in EM in the 2014 Match. The mean score for EM-bound students on the EM-ACE, V1 and V2 of the EM M4 exams were 70.9 (n=47, SD=9.0), 84.4 (n=36, SD=5.2), and 83.3 (n=11, SD=6.9), respectively. Mean scores for non-EM-bound students were 68.0 (n=256, SD=9.7), 82.9 (n=243, SD=6.5), and 74.5 (n=13, SD=5.9). There was a significant difference in mean scores in EM-bound and non-EM-bound student for the EM-ACE (p=0.05) and V2 (p<0.01) but not V1 (p=0.18) of the National EM M4 examination.

**Conclusion:**

Students who successfully matched in EM performed better on all three exams at the end of their EM clerkship.

## INTRODUCTION

Assessment using a high stakes examination is an important component of a medical student’s rotation grade. In the latest State of the Clerkship survey, on average, 25% of a student’s grade is determined by a high stakes end-of-rotation examination score. 1 Clerkship directors frequently use the National emergency medicine (EM) fourth year medical student (M4) examination or the National Board of Medical Examiners (NBME) EM Advanced Clinical Examination (ACE) for this assessment. 2–4 These examinations are administered in both required and elective rotations, thus are given to both “career-bound” (i.e. students interested in matching in EM) and “non-career-bound” students.

In addition to providing students and clerkship directors feedback on a student’s knowledge base, these examinations provide feedback on how students compare to their peers nationally. Versions 1 (V1) and 2 (V2) of the National EM M4 exams have historic means and standard deviations for examination administrations ( www.saemtests.org ), while the NBME has reported scaled scores for the EM-ACE since October 2013 (and retrospectively reported them for examination administrations before October 2013). 4 While the examination means and standard deviations vary slightly year to year, the most recent (2014–5) EM M4 V1 mean is 81.5 (SD=3.7) and V2 is 78.4 (SD=4.4). The EM-ACE is scaled to a mean score of 70 (SD=8). 5

The National EM M4 exams report statistics on the entire population of students who have taken the examination, and the EM-ACE has been scored based on all fourth-year first-time LCME-accredited medical student administrations. Little is known about how students who ultimately match in EM perform on these examinations as compared to their non-EM-bound peers.

The objective of this study was to determine the mean and standard deviation performance of students who matched in EM on the three commonly used exams for student assessment of EM medical knowledge. Additionally, we compared performance of EM-bound and non-EM-bound students on these examinations.

## METHODS

We performed this multicenter, retrospective, cohort study as a subset analysis across five U.S. allopathic medical schools between May 2013 and April 2014. During this period, the NBME was attempting to validate the EM-ACE quickly in order to develop scaled scores and the exam was offered free of charge. In order to correlate EM-ACE performance to exams that already had established validity, all fourth-year medical students participating in a fourth-year EM rotation at the study sites were administered both the NBME EM-ACE and one version of an EM M4 examination. 6 The dataset used for this study was derived from the data collected for the EM-ACE National EM M4 correlation project.

The study sites varied with regard to having mandatory selective or elective EM rotations, but were all four weeks in duration and used the standardized curriculum recommended by the Clerkship Directors in EM (CDEM). Study sites administered either V1 or V2 of the EM M4 examination based upon site preference. Exams were taken consecutively, within one day of each other, at the end of the rotation. Individual study sites determined which examination was administered first. Both exams were administered by the same clerkship coordinator or other administrator according to respective protocols developed by the NBME and CDEM. At all sites, students were aware that the EM M4 examination would count towards their grade, as per local institution protocol. Without longitudinal performance data or norms, most sites did not count NBME examination towards the final rotation grade; however, to encourage students to take the NBME examination seriously, some institutions advised students that although the NBME examination could not lower their grade, a strong performance would be reflected in their final evaluation. One institution used the NBME score for a small portion (5%) of the final course grade.

De-identified data were collected by the clerkship director or coordinator, and included institution, NBME scaled score, the version of the EM M4 examination administered (V1, V2) and the score on that examination. After the 2014 National Resident Matching Program (NRMP) Match, whether the student matched in EM (match status) was also collected as a dichotomous variable. Student’s t-tests were performed on the examination averages of students who matched in EM as compared with those who did not.

We performed data collection in Microsoft Excel 2007 and data analysis with StataMP 11.0 (College Station, TX).

This project was determined to be exempt from human subjects review by the institutional review boards of all participating institutions.

## RESULTS

A total of 606 students took both the EM-ACE and one of the versions of the National EM M4 examination. Of the total cohort, 94 (15.5%) matched into EM in March 2014. This represents 5.3% of all the EM residency positions in the 2014 NRMP Match.^7^

Students who matched in EM had higher examination averages on all three examinations. This difference was statistically significantly for the EM-ACE and Version 2 of the National EM M4 examination (p=0.05, p<0.01 respectively). See Table.

## DISCUSSION

While it is perhaps not surprising that EM-bound students perform better on EM exams than non-EM bound students, this phenomenon has not been described. We report on a small but geographically diverse sample of students who took these exams for the first time. To our knowledge, this is the first time examination means and statistics have been specifically reported for the group of students matching into EM.

Such information is valuable to students, advisors and program directors. Students should know how they score in relation to their peers, especially the cohort of EM-bound students, as this information may have a significant impact on their application, interview and match-list strategy. Additionally, clerkship directors and other medical student advisors may be able to use this information to give students an idea of how successfully matched EM-residents performed on their end-of-rotation examination. Finally, this information is valuable to program directors as an objective measure of a candidate’s EM knowledge foundation, and may predict future success on other high stakes exams, such as the American Board of EM (ABEM) in-training examination or qualifying certification examination.

## LIMITATIONS

Although the study population was taken from five geographically diverse sites, the number of students who matched in EM in this sample was small, a total of 94. This represents 5.3% of all EM spots in the 2014 NRMP match. Match status rather than interest in EM was used to identify the cohorts in part because match status is a discrete and unambiguous variable. The non-EM group consists almost entirely of students who electively pursued specialties other than EM, however, it is likely a small number of students who attempted but were unsuccessful in the EM match are included in this group. We were unable to quantify the number of students in this cohort, as intended specialty match information is only available to the applicant, and may change over time. Additionally, in advising students interested in matching in EM, exam performance compared to successfully matched applicants is a more valuable metric than performance compared to all students attempting to match in EM. Prospective collection of information about intended career goals in relation to examination performance may represent an avenue for future research.

Student scores were likely affected by the perceived importance of the examination. EM-bound students may have prepared more intensely compared to their non-EM peers due to a perceived greater impact on their future career. Sites varied as to whether the clerkship was required, selective or elective. It is possible that non-EM-bound students in an elective/selective rotation might differ from those in a required rotation, in regards to motivation and interest in EM-related material. In addition, site directors used the scores from these exams differently. While students each took the EM-ACE and one of the EM M4 exams, the EM-ACE examination constituted 0–5% of the final rotation grade, and the National EM M4 exams up to 25%. Finally, students in the EM-bound group may have had more experience in EM than their non-EM-bound counterparts prior to their examination.

It is important to note that a knowledge assessment examination is only one measure of student performance. While all these exams are high quality, high stakes, validated exams, they report on only one dimension of a student’s capacity to provide EM care. These results must be viewed as one component of the entire application when evaluating a student for residency candidacy.

## CONCLUSION

Students who matched into an EM residency performed significantly better on the NBME EM-ACE and Version 2 of the National EM M4 exams. As an objective measure of EM knowledge, these exams may help clerkship directors counsel students about their likelihood of matching into EM. Program directors may be interested in using this information in the evaluation of EM applicants.

## Figures and Tables

**Table:** Difference in emergecny medicine advanced clinical examination (EM-ACE), EM fourth year medical student (M4) version 1 (V1) and version 2 (V2) examination scores by student match status.

Examination	EM student score (SD)	Non-EM student score (SD)	P value
EM-ACE (scaled score)	70.9 (9.0) N=47	68.0 (9.7) N=256	0.05
V1 EM M4 examination (percent correct)	84.4 (5.2) N=36	82.9 (6.5) N=243	0.18
V2 EM M4 examination (percent correct)	83.3 (6.9) N=11	74.5 (5.9) N=13	<0.01

*SD,* standard deviation
